# Implementation and Strategies to Support Adoption of the World Heart Federation Roadmap on Cardiac Rehabilitation: A Pathway to Improve Lifelong Cardiovascular Health

**DOI:** 10.5334/gh.1563

**Published:** 2026-07-13

**Authors:** Dion Candelaria, Irene Gibson, Abraham Samuel Babu, Tom Briffa, Lilian Mbau, Robyn Gallagher, Wen-Chih Wu, Alexis Beatty, Gabriela L. M. Ghisi, Alice Namanja, Dawn Scantlebury, Giulliano Gardenghi, Yihong Sun, Aashish Contractor, Ana Abreu, Roque Daniel González, Pat O’Donnell, Rongjing Ding, David Wood, Randal J. Thomas, Sidney C. Smith JR, Julie Redfern

**Affiliations:** 1 Susan Wakil School of Nursing and Midwifery, Faculty of Medicine and Health, University of Sydney, NSW, Australia; 2 National Institute for Prevention and Cardiovascular Health, University of Galway, Republic of Ireland; 3 Department of Physiotherapy, Manipal College of Health Professions, Manipal Academy of Higher Education, Manipal, India; 4 University of Western Australia, WA, Australia; 5 Centre for Cardiovascular Prevention and Rehabilitation, Nairobi, Kenya; 6 Cardiovascular Institute, Brown University Health, United States of America; 7 University of California, San Francisco, United States of America; 8 University Health Network, University of Toronto, Canada; 9 Kamuzu University of Health Sciences, Blantyre, Malawi; 10 The University of The West Indies, Barbados; 11 Brazilian Association of Respiratory, Cardiovascular and Intensive Care Physiotherapy, São Paulo, Brazil; 12 Beijing Anzhen Hospital, Capital Medical University, National Clinical Research Center for Cardiovascular Diseases, Beijing, China; 13 Sir HN Reliance Foundation Hospital, Mumbai, India; 14 Lisbon Medical School, University of Lisbon & ULSSM, Lisbon, Portugal; 15 Ministerio de Salud, Tucumán, Argentina; 16 Global Heart Hub, Galway, Republic of Ireland; 17 Peking University People’s Hospital, China; 18 Mayo Clinic, Rochester, Minnesota, United States of America; 19 University of North Carolina, United States of America; 20 Institute for Evidence-Based Healthcare, Bond University, Robina, QLD, Australia; 21 Faculty of Medicine and Health, University of Sydney, NSW, Australia

**Keywords:** Cardiac Rehabilitation, Secondary Prevention, Cardiovascular Disease, Heart Disease, Roadmap, Implementation

## Abstract

An ageing population means more people are living longer with cardiovascular disease. As a result, health systems face sustained pressures to provide effective long-term care. Cardiac rehabilitation has evolved from group-based exercise-based programs to comprehensive secondary prevention, with a growing focus on supporting lifelong cardiovascular health. The World Heart Federation (WHF) recently published a Roadmap that advances this shift by reframing cardiovascular rehabilitation around person-centred, future-focused care, and identifying five key recommendations: prioritise lifelong health, strengthen patient and clinician engagement, adopt new models of care, build workforce capacity, and advance advocacy and policy. This paper outlines practical implementation strategies to support adoption of the WHF Roadmap on Cardiac Rehabilitation (a pathway to improve lifelong cardiovascular health) in diverse real-world settings. We present adaptable approaches at the patient, clinician, and system levels, focusing on how cardiovascular rehabilitation can be embedded across the continuum of care with modernised concepts and terminology. Key strategies include integrating rehabilitation earlier and more consistently into care pathways, leveraging data for continuous quality improvement, and aligning funding mechanisms and policy frameworks to enable sustainable delivery. Importantly, implementation must remain responsive to local contexts, including variations in resources, workforce capacity, and population needs. Robust research strategies are also outlined that evaluate approaches to translate global priorities into routine practice and support the concept of being the lifelong cardiovascular health program for all.

## Introduction

Cardiovascular disease (CVD) remains the leading cause of disease burden and death worldwide, disproportionately affecting low, low-middle, and middle socio-demographic index regions (1). Advances in diagnostics, revascularisation, medications, and interdisciplinary care (2,3) have substantially improved survival after acute events. While these developments represent major progress, they have also contributed to an increasing proportion of people living longer with CVD, often following shorter lengths of stay in hospital (4). Concomitantly, an ageing population and the continuance of unhealthy lifestyle patterns influenced by broader social and structural determinants amplify health system pressures to support long-term CVD management (3). Stronger public health infrastructure and support for implementation are therefore needed.

Cardiac rehabilitation has long been the primary approach for delivering structured, comprehensive secondary prevention (5,6). Participation in cardiac rehabilitation has a strong evidence base and is associated with improved functional capacity, quality of life, medication adherence, and risk factor management (7,8,9,10). It also contributes to reductions in recurrent events, mortality, and hospitalisations, while consistently demonstrating favourable cost-effectiveness (11,12). Originally developed over 50 years ago as group-based exercise with education programs typically delivered several times per week over 6 to 12 weeks (6), cardiac rehabilitation has evolved to place greater emphasis on holistic physical and psychosocial recovery (5,6). Core components in such programs include clinical assessment and review, multidisciplinary care, management of cardiovascular risk factors (e.g., diet, weight, hypertension, dyslipidaemia, diabetes, smoking, physical inactivity), structured exercise training, and program evaluation (5).

Despite strong evidence and consistent recommendations across international guidelines (13,14,15,16,17), cardiac rehabilitation delivery remains variable in availability, scope, intensity, and mode with challenges associated with funding, awareness, and geography (6,18,19). Referral, participation, and completion rates remain persistently low (20), leading to calls for change that address equity, access, and better outcomes. Furthermore, initial changes to lifestyle achieved by program attendance are often not sustained, and medication adherence also typically declines within 6 to 12 months (21,22,23). Globally, many programs remain under-funded and have insufficient collection of quality-based metrics; these factors also contribute to inequity between and within countries and sub-groups that could have benefited (25). Indications for these programs should be broadened to ensure they are inclusive of all individuals living with CVD (including but limited to the concept of panvascular prevention) (24). Taken together, current evidence-practice gaps underpin a need to reimagine traditional cardiac rehabilitation into an integrated cardiovascular health program that supports lifelong cardiovascular well-being for all who can benefit (3) To advance the field, there is therefore growing momentum to move beyond time-limited and group-based programs by implementing structures and programs that support individuals and families over the longer term to live healthier lives.

In response to this need, the World Heart Federation (WHF) published a Roadmap on Cardiac Rehabilitation that presents a united vision to advance efforts that enhance equitable access, improve clinical outcomes, and ensure value-based healthcare (26). Processes and methods for the developing the WHF Roadmap are detailed elsewhere (26). In brief, an iterative process was followed that included a global survey, an in-person Global Cardiac Prevention and Rehabilitation Forum (i.e., the Global Forum) held on 29 August 2024 in London, and the development of an associated Roadmap by an international Expert Writing Group (26). Ethical approval for the survey and forum was obtained from the University of Sydney, Human Research Ethics Committees (2024/HE000426).

Together, the online survey and the Global Forum (52 global experts and emerging leaders from 20 countries, Supplement 1) enabled the collection of perspectives on the challenges in delivery of and engagement with cardiac rehabilitation programs globally, as well as possible solutions to improve program reach and uptake. Results identified several priority areas: the need for global standards; strengthening of the workforce; more flexible, person-centred models of care; and greater awareness of and advocacy for cardiovascular health programs among patients, clinicians, and policymakers (26).

The published WHF Roadmap on Cardiac Rehabilitation (hereinafter referred to as the Roadmap) (26) aligns with previous WHF roadmaps that addressed secondary prevention, digital health, and hypertension (27,28,29). Its solutions form part of the WHF commitment to improving global cardiovascular health and contribute to the United Nations Sustainable Development Goals (30). The Roadmap is endorsed by the International Council of Cardiovascular Prevention and Rehabilitation (ICCPR) and proposes a revision of the 1993 World Health Organization definition (31) of cardiac rehabilitation (Box 1). The new definition places greater emphasis on continuity of care, considering each person’s journey toward lifelong cardiovascular health. Whereas the 2026 WHF Roadmap establishes a global strategic vision for reorienting cardiac rehabilitation toward lifelong cardiovascular health, the present paper complements that vision by addressing how change can be operationalised in practice. Specifically, it proposes actionable implementation strategies with illustrative examples along with future research priorities that support uptake, adaptation, and sustainability across settings.

**Box 1: Redefining ‘cardiac rehabilitation’ programs as ‘cardiovascular health’ programs (26)**

‘The systematic provision of ongoing care and support for people with, or at elevated risk of, CVD, that in combination with all aspects of evidence-based prevention and rehabilitation will lead to lifelong cardiovascular well-being. Care should be person-centred and encompass all aspects of lifestyle optimisation, clinical management of risk factors, psychosocial support, and adherence to guideline-directed medical therapy.’

## Overview of Roadblock Solutions

**Figure 1:**
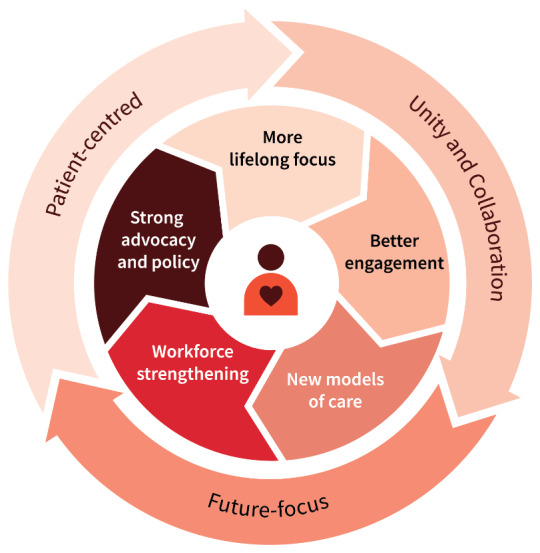
Schematic overview of proposed solutions to the five interlinked roadblocks.

Five interconnected roadblocks and corresponding solutions were identified and are detailed in the published the Roadmap (26), which reframes terminology and solutions to be grounded in unity, person-centredness, and future-focused thinking; and is designed to be adaptable across diverse contexts and resource settings (Figure 1). The five key solutions are: (1) emphasizing focus on lifelong cardiovascular health, (2) strengthening engagement among patients and clinicians, (3) expanding new models of care, (4) strengthening workforce capacity, and (5) supporting advocacy and policy (Figure 1). The solutions are framed around the previously published 5Ps for reframing cardiac rehabilitation, which are personalisation, processes, person-centred care, parlance, and partnership (3). The Roadmap also includes five case studies from Malawi, Barbados, India, Argentina, and the Republic of Ireland (26). Below is a summary of the roadblocks and solutions, along with additional case studies.

### Solution 1: Emphasising focus on lifelong cardiovascular health

The term *cardiac rehabilitation* originated when recovery after myocardial infarction was prolonged, hospital-based, and diagnosis-specific, making a finite ‘rehabilitation’ period appropriate. The focus has been on a finite period of recovery following isolated ‘cardiac’ events rather than on achieving lifelong cardiovascular health for people living with a wide range of cardiovascular conditions, including peripheral and cerebrovascular diseases (3,24,26).

Potential solutions presented in the Roadmap reframe cardiac rehabilitation as a cardiovascular health program (26). This reflects a shift toward ongoing, person-centred care in which cardiovascular health programs are the entry point for lifelong self-management (3) across all manifestations of CVD (16). This broader concept expands eligibility, reinforces consistent messaging about sustained health rather than time-limited rehabilitation, and encourages continued support beyond program completion (3,26,32). Such support may include links with community services, peer and family involvement, cultural health workers, long-term follow-up, and use of digital tools. In addition, embedding cardiovascular health programs within chronic disease management policies alongside scalable community and system-level interventions will reinforce and strengthen program benefits and impact. Targeted communication and engagement strategies for patients, clinicians, and policymakers will also help strengthen program reach, relevance, uptake, and sustainability by providing each group with clear, tailored information that supports participation and decision-making.

### Solution 2: Strengthening engagement among patients and clinicians

The period that immediately follows a cardiovascular diagnosis or acute event represents a critical window of opportunity (33), yet many patients are not identified, referred, or encouraged to engage in programs that could improve long-term cardiovascular health and reduce future risk (20). Several factors are at play, including shorter hospital stays, inconsistent referral processes, and the absence of integrated electronic systems, which limit timely education and discharge planning. Patient-level factors, such as language, culture, health literacy, and comorbidities further complicate engagement during the acute care period (34,35). The strength of clinician referral also varies widely, often due to time constraints, limited awareness of program benefits, and lack of clear responsibilities within the care team (36,37,38).

Solutions emphasize early, systematic, and shared responsibility for referral, and education for both patients and clinicians. Face-to-face clinician engagement with patients and families during hospitalisation, supported by clear and culturally appropriate messaging has been shown to be effective (37). Structured pathways with automated referral processes ensure systematic referral and enrolment rates and reporting to incentivise consistent practice (38,39). Workforce capacity building including task-shifting to nursing and allied health staff and stronger integration between inpatient and outpatient services are essential (40,41). Ongoing clinician education including about the value of guidelines, use of telehealth and flexible delivery is also valuable. Patient awareness should be reinforced through tailored educational materials, complemented by community outreach and public messaging to engage diverse and underserved populations (35,42,43).

***National Audit of Cardiac Rehabilitation (NACR), UK*** (44). Since 2005, the National Audit of Cardiac Rehabilitation (NACR) has produced regular reports on cardiac rehabilitation service quality, uptake, and outcomes in England, Wales and Northern Ireland at local, regional and national levels for the National Health Service (NHS). Since its inception, the NACR has facilitated quantifiable improvements in service provision and has been a key part of the UK Government Research Excellence Framework. The audit promotes best practice and improves quality in cardiovascular prevention and rehabilitation services by:

- Examining reasons for variation in clinical outcomes between programs as a resource to help services improve using quarterly reporting, via NHS online platforms;
- Sharing national trend data with appropriate national bodies including the Department of Health, NHS England, National Institute for Clinical Excellence, Cardiovascular Care Partnership UK and the British Association for Cardiovascular Prevention & Rehabilitation (BACPR); and
- The National Certification Programme for Cardiac Rehabilitation (NCP_CR), which aligns with BACPR Standards and Core Components. In 2025, 53% of programs met all the key performance indicators and have been awarded ‘Green’ certified status.

### Solution 3: Expanding new models of care

Low uptake of traditional cardiac rehabilitation programs reflects a combination of well-known individual, environmental, logistical, and systemic barriers. Factors such as older age, gender, multiple comorbidities, low motivation, limited social support, rural location, travel distance, scheduling constraints, cost, insurance coverage, and inconsistent referral processes have all been found to limit participation (35,45,46,47,48). At a system level, fragmented care pathways, limited resources and funding, and inadequate technology infrastructure constrain the ability to deliver personalised care. The barriers are exacerbated in low-resource settings and in low- to middle-income countries (8,19,25,38). Addressing these challenges requires a shift toward greater flexibility, personalisation, and process improvement (3,26).

**Figure 2:**
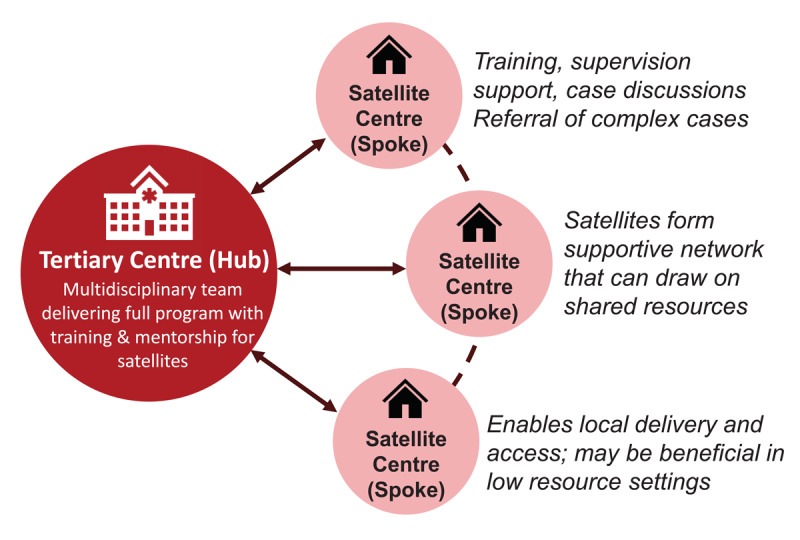
Hub-and-spoke model for cardiovascular health programs.

Solutions should prioritise person-centred, flexible models of care that embrace lifelong cardiovascular health (3,26,49). Programs should promote availability of choice for participants, be multidisciplinary (50), culturally adapted, and target the full range of risk factors, including psychosocial health and social determinants (3,51,52,53,54). Collection of routine data with feedback loops is important to ensure responsiveness to the needs of all participants and to enable continuous improvement. Flexible delivery formats such as telehealth, home-based, digital, and hybrid models (16,51,55,56,57) can improve engagement when core components and workforce allow, although health and technological literacy must be considered (58). Emerging models of care can also extend reach while maintaining quality and efficiency with standardisation (assessment, education, personalised care planning, follow-up) and inbuilt flexibility, such as the Secondary Prevention for All in Need (SPAN) framework (59). This model provides a framework where low and high resource settings can implement a minimal viable and evidence-based approach with broadening and strengthening where possible (59). One example of an alternative structure is the hub-and-spoke model (60) in which large tertiary centres (hubs) support resource-limited regional sites (spokes) (Figure 2).

***Case Study: Integration of CVR into primary care frameworks, China***. The growing burden of CVD and an ageing demographic in China has driven increasing demand for rehabilitation services. However, service availability and accessibility remain limited, particularly in rural and underserved regions. To address these gaps, the China Society of Cardiopulmonary Prevention and Rehabilitation has:

- Established a national registry system while implementing specialised workforce training programs and evidence-based guidelines.
- Integrated rehabilitation frameworks into primary care with strengthened specialist referral pathways to ensure timely, cost-effective service delivery.
- Updated their national guidelines to standardise cardiopulmonary rehabilitation protocols for community implementation, with remote monitoring and artificial intelligence (AI) clinical support being piloted in resource-limited areas.

### Solution 4: Strengthening workforce capacity

The effective delivery of comprehensive cardiovascular health programs depends on a qualified multidisciplinary workforce with expertise in cardiovascular care, behaviour change, exercise, nutrition, psychosocial well-being, and pharmacotherapy (5). Workforce capacity is influenced not only by the health system structure, but also by staff turnover, evolving evidence and technologies, and variable levels of expertise; ongoing training and capability building are therefore essential. Several barriers to building a strong qualified workforce include geographical constraints, limited standards and recognised continuing professional development pathways, insufficient data to inform workforce planning, and chronic underinvestment in prevention-focused roles compared with acute care.

**Figure 3:**
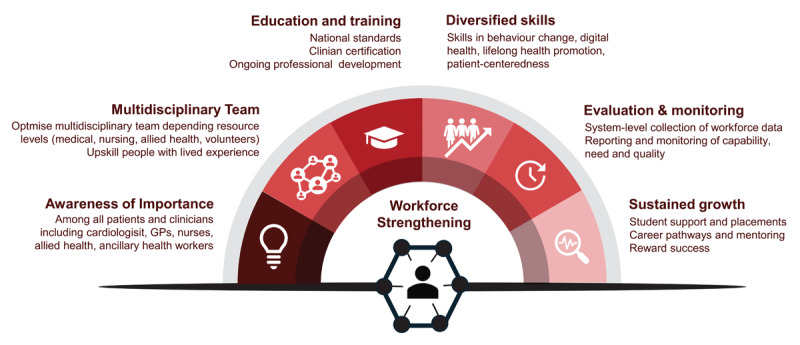
Strategies for strengthening the cardiovascular health program workforce.

Solutions for strengthening workforce capacity require sustained system-level investment in education, skills development, evaluation, and long-term growth (Figure 3). Raising awareness of the value of cardiovascular health programs among clinicians and patients is also critical (26). Multidisciplinary teams should be optimised to local resources, incorporating medical, nursing, allied health, community health workers and potentially trained volunteers supported by clear referral pathways (5). Health systems play a key role in establishing national training standards, certification processes, and ongoing professional development to ensure fundamental competencies while adapting to evolving evidence and models of care. Workforce capability should encompass clinical expertise alongside skills in behaviour change, digital health, and person-centred care. Continuous monitoring through accessible data systems can inform planning and improvement (61,62,63), while sustained growth depends on investment in training, mentorship, and career development to support a resilient and equitable workforce (26).

***Case Study: National roll-out of standardised minimum clinician certification, Australia***.

- Australia has a nationwide collaborative program with ICCPR (64), funded by their national association (Australian Cardiovascular Health and Rehabilitation Association, ACRA) and supported by the SOLVE-CHD program to ensure programs have at least one staff member with minimal/foundation qualifications.
- In the first year since introduction, over 375 clinicians have completed the ICCPR Cardiac Rehabilitation Foundations Certificate, with a target set of over 50% of Australian programs having at least one clinician certified.

### Solution 5: Supporting advocacy and policy

Advocacy and policy ensure that participation in cardiovascular health programs is recognised as an essential aspect of long-term management for all people with CVD (3). However, existing advocacy and policy efforts are often fragmented, are insufficiently aligned across patient, clinician, and system levels, and are not future-focused. Stronger partnerships and unified advocacy are needed to elevate cardiovascular health programs within national and global agendas, strengthen political commitment, and support sustainable investment in prevention and long-term care.

Solutions focus on effective advocacy with a coordinated voice that aligns with local and global priorities (26). Positioning cardiovascular programs as accelerators of international commitments, such as the UN Sustainable Development Goals (30) and the WHO Rehabilitation 2030 Initiative (65), strengthens engagement, funding, and reform. Critically, involving people with lived experience through advocacy training and accreditation amplifies consumer voices (3,26,66). Robust individual- and system-level data, endorsed by professional societies and policy stakeholders, further reinforce these efforts. Integrating lifelong cardiovascular health into clinical guidelines—via person-centred, culturally adapted, and flexible models—is essential for resource prioritisation. Finally, clear vision and evidence-informed lobbying can drive investment and universal health coverage, particularly in low-resource settings where contextualised guidance and data-driven advocacy are vital for success (26).

***Case study: Successful government lobbying, Cameroon/Senegal***. These governments were successfully lobbied to reimburse cardiovascular health programs that demonstrated a commitment to improving healthcare financing and access, creating an environment conducive for advocating the reimbursement of cardiovascular health programs.

- In Cameroon, there was prioritisation of frontline financing, including cardiovascular health programs, to improve equity in spending and expand primary care.
- In Senegal, a national health financing strategy was established to achieve universal health coverage that included cardiovascular health programs.
- Promotional resources featured in scientific publications were demonstrated to serve as important advocacy documents that highlighted the importance of resourcing for cardiovascular health programs.
- Collection and presentation of real-world data was essential to advocacy success.

## Strategies to Support Implementation

**Figure 4:**
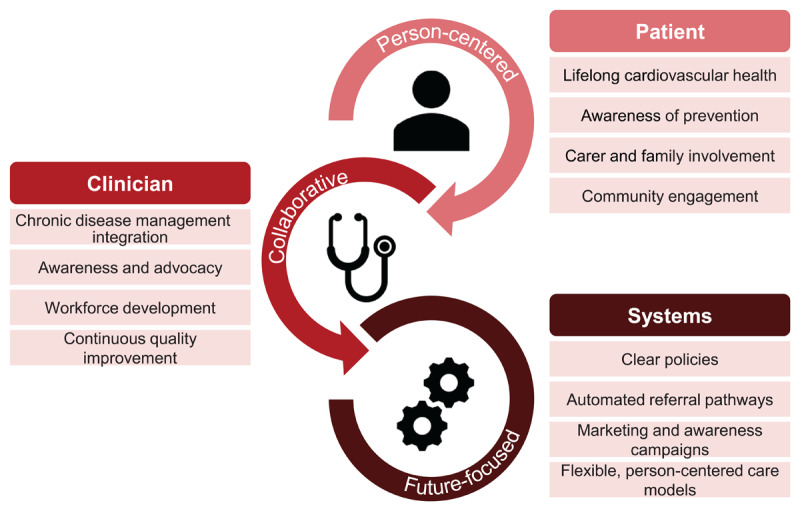
Multi-level implementation strategies for high quality cardiovascular health programs.

The proposed solutions to identified roadblocks are vital to enhance the uptake of programs and promote lifelong cardiovascular health, and ensure adaptability to local contexts and flexibility for different settings (26). This endeavour becomes even more vital in low- and middle-income countries, where program availability is suboptimal (8,18–19). Developing strategies to address these roadblocks across economic and cultural settings presents a unique opportunity for intersectoral collaboration and fosters innovation in implementation and research design. This section describes a series of implementation strategies at the patient-, clinician- and system-levels based on implementation science and quality improvement models with an underlying premise of being person-centred, collaborative and future-focused (Figure 4).

### Patient-level implementation strategies

A variety of patient-level strategies could be implemented into cardiovascular health programs to address the identified roadblocks. These include ensuring each person participating in a program has a personalised prevention plan that addresses long-term functional and psychosocial well-being (3,26). Provision of personalised cardiovascular health plans (which could be termed ‘cardiovascular health passports’) could facilitate lifelong cardiovascular health by supporting patients to track their risk factors, goals, and strategies to control them. Such documents (hardcopy or digital) could be maintained by the patient to empower them to self-manage their cardiovascular health in the long-term. Cardiovascular health and ‘stamps’ could be provided during health check-ups to incentivise achievement of goals and to remind patients about actions for future consultations. Importantly, offering flexible delivery modes (e.g., facility, community, home, digital, hybrid options) matched to individual clinical risk, preference, and capability also facilitate access to person-centred cardiovascular health (3,26). Wearable devices and mobile health technologies may further support these strategies by enabling continuous monitoring of physical activity, heart rate, and other risk factors, and by providing timely feedback and motivation for patients and clinicians (67).

To promote awareness of prevention and involvement, availability of and support for lived experience champions and peer support groups could be used to foster sharing of lived experiences, check-ins and encouragement to attend programs and continue lifelong prevention (68). Further, involvement of carers and family members and advocacy via community-led groups (e.g., sporting, cultural, worship groups) can be excellent ways for promoting awareness of cardiovascular health programs and for encouraging health and well-being for people living with CVD. Person-centred approaches to implementation also include facilitating access to guidelines and their recommendations through lay language and visual summaries of cardiovascular health information (69,70). Production and sharing of lived experience testimonials, advisory groups, and collection and integration of patient experience measures could also help to overcome roadblocks to person-centred lifelong cardiovascular health (3).

### Clinician-level implementation strategies

Clinician training and program quality are important drivers of strengthening cardiovascular health programs. Implementation strategies include ensuring all levels of clinician training feature an awareness of the importance of cardiovascular health programs (26,41). Such education should be embedded in undergraduate education (including student placements) for all clinicians, including medical, nursing, allied health workers and community health workers (26). This will help to ensure a pipeline of trained personnel. Once practicing, ongoing training and support for clinicians should ideally include competency checks, standardised certification, and availability of specialised research and postgraduate education programs (26,38,64). Importantly, awareness of evidence for cardiovascular health programs should be integrated across medical practices, including among general practitioners, cardiologists and surgeons, because all clinicians can have a positive influence of patent participation in programs.

Integrating clinician training and awareness into chronic disease management opportunities can also facilitate better engagement with cardiovascular health programs. This can include mentorship and cross-disciplinary awareness and skills-based strengthening. Train-the-trainer and mentorship programs embedded in hub-and-spoke models could be a valuable strategy for facilitating clinician capability in rural and low-resourced settings (60). Clinician-level strategies should not only consider knowledge about risk factor management but should also include awareness and training in facilitating self-management, flexibility in program offerings, digital and hybrid communication and care, as well as advocacy and communication, with lifelong cardiovascular health being the underlying goal (3,26). Further, clinicians should be supported to facilitate shared decision-making to foster the ongoing trust and engagement of program participants (71).

Collaborative strategies among multidisciplinary clinicians could include task sharing/shifting with training of alternate team members to deliver aspects of care, ensuring all components of cardiovascular health programs are delivered using all available resources (72). Collaboration with professional organisations could facilitate such continuing professional development. For example, the Heart Failure Association of India has introduced online programs to upskill nurses and physicians while yoga physicians (a traditional system of medicine in India) are being trained through a virtual certification program in Yoga-based cardiovascular health programs to enhance cultural relevance (53). A further example is in Australia, where enabling access to Aboriginal Health Worker and/or Liaison Officers and a clinician buddying system have been introduced to overcome suboptimal program engagement by Indigenous Australians (73). Other practical strategies to facilitate task-sharing include the availability of toolkits, resources, and factsheets to facilitate treatment planning, medication, and lifestyle adherence (74,75).

### System-level implementations strategies

Strengthening cardiovascular health programs requires clear policy direction and coordinated system leadership (26). This includes redefining programs to support lifelong cardiovascular health, and broader clinical indications through collaboration among professional bodies, policymakers, insurers, and lived experience representatives (76). Programs should align with national non-communicable disease strategies and integrate with primary care and public health services targeting key risk factors. Policy approaches can also support competency-based training, minimum prevention bundles, and national indicators to guide implementation. Building coalitions across professional societies, government agencies, consumer groups, and implementers—supported by investment cases demonstrating value—can facilitate scale-up and financial accessibility (26). Examples include the Million Hearts Initiative (33), the European Union Cardiovascular Health Plan (Safe Hearts Plan) (77), and the Australian Core Components of Cardiovascular Rehabilitation Position Statement (78), which advocate for national CVD plans delivering high-quality, multidisciplinary care.

Health system-level and policy implementation strategies should aim to improve access to cardiovascular health programs and facilitate integration across chronic disease management and primary care pathways with consideration of transport, fees, and workforce capability. Implementation of a flexible framework, such as SPAN, enables flexibility within and between programs where low resource settings could deliver a minimal viable program and high resource settings could implement a broader and longer-term approach (59). This is a tiered approach within programs, based on the needs and preferences of participants as well as between programs approaches based on capacity and resourcing. Any evaluation of such an implementation should consider the needs of all those with multiple risk factors who are at high risk of CVD, thereby embracing the concept of panvascular prevention with a focus on cardiovascular health (24,79).

Implementation of flexible models with systematic referral pathways are critical to improving program enrolment and continuity of care. For example, automated referral for all eligible people using electronic triggers, checklists, and prompts can help ensure no person is missed, with flexible options made available to program participants based on their needs and preference (38,39). Referral pathways should operate across the care continuum, including pre-procedure enrolment and post-discharge follow-up. Data systems, dashboards, and patient-reported outcomes can further support monitoring and continuous improvement (10,61,63,80). Use of plan-do-study-act cycles (81) can offer practical ways for programs and systems to monitor, benchmark and set targets for optimising quality (61).

Advocacy and media campaigns, along with community events, can also be valuable in increasing public engagement and promoting lifelong cardiovascular health. Examples include strategies used by (i) the Million Hearts Initiative, which promotes advocacy through partnerships (33), public awareness campaigns and highlighting success stories; and (ii) the WHF Beats of Change initiative, which included a feature on a lived experience story ‘Life After Heart Disease’, produced by BBC Storyworks (82). Partnerships with community leaders, urban designers, administrators and stakeholders are also vital to help redesign communities with cardiovascular health in mind (26). Creating active societies through community engagement are vital to scaling up the ParkRun and HealthPark initiatives (83,84).

## Implementation Research Opportunities

Research is integral to advance knowledge on evidence-based solutions for implementing CVD health programs. In addition to examining clinical and patient-reported outcomes, research should also prioritise implementation outcomes such as acceptability, adoption, appropriateness, feasibility, fidelity, implementation cost and sustainability as key endpoints (85). Research studies with a focus on implementation outcomes are important for generating evidence on the most effective strategies for overcoming barriers to CVD prevention in a given context (86). Table 1 presents an overview of potential implementation strategies mapped to Expert Recommendations for Implementing Change (ERIC) (87) classifications with discrete actions and potential evaluation methods.

**Table 1:** Overview of potential strategies, actions and evaluation to implement the WHF Roadmap on Cardiac Rehabilitation (pathway to lifelong cardiovascular health) using the Expert Recommendations for Implementing Change (ERIC) framework (87).


STRATEGY	MAPPING TO ERIC CLASSIFICATION	DISCRETE ACTIONS	EVALUATION METHODS

**Patient-level**			

Cardiovascular health plans (could be termed ‘cardiovascular health passports’)	Develop and organise quality monitoring, use clinical data systems, promote patient-held records	Develop and implement tools, integrate into care pathways, provide patient education and tools	Audit and feedback, quality improvement cycles, attendance, clinical outcomes, satisfaction

Patient education and engagement strategies	Conduct educational meetings, distribute educational materials, involve patients and family members	Deliver tailored education materials, peer support programs, community outreach	Pre–post evaluation Patient-reported outcomes

Access to person-centred and flexible models of care	Change service sites, revise professional roles, promote adaptability	Offer different models of care and modes of delivery with suitable workforce capability and resources	Reach and completion of programs, diversity of attendance, patient satisfaction

**Clinician- and program-level**			

Workforce certification and training programs	Conduct educational meetings, develop educational materials, create a learning collaborative	Training and skills development, certification pathways, continuing education programs	Workforce competency metrics, uptake rates, clinician awareness

Integration into clinical pathways	Change record systems, revise professional roles, promote integration of services	Embed programs into primary and hospital care planning and chronic disease management	Cluster and stepped-wedge trials with evaluation of process, cost and patient-reported measures

Automated referral systems	Change record systems, facilitate relay of clinical data to providers, use clinical decision support	Implement electronic or paper-based referral triggers, training for staff	Audit and feedback, referral/enrolment/completion rates, eligible patient access

**System-level**			

Universal access to lifelong cardiovascular health	Use policy levers, develop stakeholder interrelationships, promote adaptability, alter incentive structures	Policy integration, national rollout, standardisation	Policy evaluation with population-level indicators and unified data systems

Global benchmarking and registries	Audit and feedback, develop and organise quality monitoring systems, use data experts	Establish registries, standardise indicators based on evidence, enable cross-country comparisons	Observational analyses, multinational studies, case studies, wide dissemination

Mass media and awareness campaigns	Use mass media, conduct local consensus discussions, involve patients/consumers	Public campaigns, targeted messaging	Population awareness metrics, attendance and completion rates, health outcomes, value


Future research could evaluate innovative models of care to better understand varied delivery (rather than a one-size-fits-all) approaches, long-term effectiveness, safety and cost-effectiveness (3,26). This could include comparing models that prioritise inclusion of diverse population groups including women, older adults and under-represented ethnic minority groups (35,40,41,45,46,47). Adequately powered non-inferiority/equivalence study designs could be used, and for a more pragmatic approach, existing data registries could enable comparisons between delivery models within and across programs (26,88). Importantly, co-design methods should be used to support person-centred adaptation and ensure interventions are developed to meet the needs and expectations of the target population, including their being culturally responsive. Co-design and implementation testing of personalised ‘cardiovascular health passports’ could facilitate lifelong cardiovascular health where patients themselves track progress, goals and strategies with clinician and health system support and facilitation.

The use of robust study designs will increase the likelihood of successful implementation and translation into practice. This includes large-scale randomised controlled trials (RCTs) or cluster RCTs with objective outcomes. Stepped-wedge and other hybrid implementation trial designs could also evaluate generalisability and translation into practice (86). Ideally, such studies should follow well-accepted frameworks such as RE-AIM (89). Research studies should also include process evaluations as well as cost effectiveness and cost of implementation in different settings (86). This will ensure a detailed understanding of barriers and accelerators to implementation from varied perspectives and contexts (86). Leveraging large international registries could strengthen cohort studies with benchmarking of targets and facilitate site-specific quality improvement initiatives. Such registries offer routinely collected representative health data and ideally should capture comprehensive clinical, behavioural, and contextual information with data consistency across jurisdictions (88).

While there is growing evidence for the use of digital interventions in cardiovascular care, including AI, few are deployed at scale and there is a lack of data on strategies and approaches for successfully integrating use of AI-based modelling studies into real-world clinical practice. Acknowledging the fast-paced evolution of these technologies, clinical research trials with a dual focus on effectiveness and implementation—for example hybrid effectiveness-implementation trials—are required (90). It is suggested that the design of these trials consider three domains: the digital intervention (software such as website or mobile aps); the clinical support services required; and implementation strategies (methods and techniques that enhance the adoption, integration and sustainment of the clinical intervention/practice) (91). To provide a structured, visual framework to help plan, execute, and evaluate implementation, the Implementation Research Logic Model can be used (92).

## Conclusions

Effective implementation is critical to ensure the WHF Roadmap on cardiac rehabilitation (a pathway to improve lifelong cardiovascular health) is translated into practice and achieves meaningful impact. Strong, sustained engagement from the WHF, WHO, and national and local champions is essential to convert its recommendations into policy reform, service redesign, and long-term investment in systems that deliver lifelong cardiovascular care. The new Roadmap offers a deliberate and decisive reframing of terminology and an expanded scope that includes all manifestations of CVD, supported by concrete, adaptable solutions and case studies that demonstrate feasibility across diverse contexts. Embedding implementation strategies into practice requires coordinated planning, workforce development, and measurement systems that incentivise equitable, high-value care. Central to this effort are authentic patient partnerships, interdisciplinary collaboration, and unified sector leadership. Future research should evaluate implementation across varied settings to identify the enablers, barriers, and system conditions that shape successful adoption. Launching a unified global cardiovascular health awareness campaign, led by WHF with a focus on lifelong cardiovascular health, will increase widespread engagement with and understanding of programs.

## Additional File

The additional file for this article can be found as follows:

10.5334/gh.1563.s1 Supplementary Material. Participants in the 2024 Global Cardiac Prevention and Rehabilitation Forum.
