# Supplementary material for: Implementation and Strategies to Support Adoption of the World Heart Federation Roadmap on Cardiac Rehabilitation: A Pathway to Improve Lifelong Cardiovascular Health
Source: Glob Heart. 2026 Jul 13;21(1):54. doi: 10.5334/gh.1563 (PMC13378420; doi:10.5334/gh.1563)
Supplement: Supplementary Material. — Participants in the 2024 Global Cardiac Prevention and Rehabilitation Forum. [file gh-21-1-1563-s1.pdf]

Supplementary Material: Participants in the 2024 Global Cardiac Prevention and Rehabilitation Forum

Helen Alexander (British Association for Cardiovascular Prevention and Rehabilitation & Association of Chartered Physiotherapists in Cardiovascular Rehabilitation, UK), Alexis Beatty (University of California, San Francisco, USA), Carmel Bourne (Australian Cardiovascular Health and Rehabilitation Association, Australia), Tom Briffa (University of Western Australia, Australia), John Buckley (International Council of Cardiovascular Prevention and Rehabilitation & Keele University, UK), Susimeire Buglia (Brazilian Society of Cardiology, Brazil), Dion Candelaria (University of Sydney, Australia), Sophie Charles (Irish Association of Cardiac Rehabilitation, Republic of Ireland), Hung-Jui Chuang (Taiwan Academy of Cardiovascular and Pulmonary Rehabilitation, Taiwan), Susan Dawkes (British Association for Cardiovascular Prevention and Rehabilitation & Robert Gordon University, UK), Diann Gaalema (American Association of Cardiovascular and Pulmonary Rehabilitation & University of Texas, USA), Robyn Gallagher (University of Sydney, Australia), Sandra Ganly (National Institute for Prevention and Cardiovascular Health, Republic of Ireland), Giulliano Gardenghi (Brazilian Association of Respiratory, Cardiovascular and Intensive Care Physiotherapy, Brazil), Sarah Gauci (Deakin University, Australia), Gabriela Ghisi (International Council of Cardiovascular Prevention and Rehabilitation, Canada), Irene Gibson (National Institute for Prevention and Cardiovascular Health & University of Galway, Republic of Ireland), Martha Gulati (American Society for Preventive Cardiology, USA), Sally Hinton (British Association for Cardiovascular Prevention and Rehabilitation, UK), Karice Hyun (University of Sydney, Australia), Alun Jackson (Australian Centre for Heart Health, Australia), Catriona Jennings (National Institute for Prevention and Cardiovascular Health & Association of Cardiovascular Nursing and Allied Professions, Republic of Ireland), Neil Johnson (Global Heart Hub, Republic of Ireland), Jennifer Jones (National Institute for Prevention and Cardiovascular Health & University of Galway, Republic of Ireland), Shanice Jones (Heart and Stroke Foundation of Barbados, Barbados), Kornelia Kotseva (National Institute for Prevention and Cardiovascular Health & Imperial College Healthcare NHS Trust, UK), Lisa Maher (Preventive Cardiovascular Nurses Association, USA), Warner Mampuya (Association Francophone de Cardiologie Preventive, Canada), Lela Maskhulia (Georgian Association of Cardiac Prevention and Rehabilitation, Georgia), Sheona McHale (Edinburgh Napier University, UK), Richard Mindham (European Society of Cardiology, UK), Alice Namanja (Kamuzu University of Health Sciences, Malawi), Josef Niebauer (Austrian Working Group on Out-Patient Cardiac Rehabilitation & Austrian Society of Prevention and Rehabilitation, Austria), Julia Ning (University of Sydney, Australia), Tone Norekval (Haukeland University Hospital, Norway), Deirdre O'Reilly (Saolta University Health Care Group, Ireland), Alexandria Palma (Scientific Society of Kinesiology in Cardiology and Cardiac Surgery of Chile, Chile), Gianfranco Parati (World Hypertension League, Italy), Trond Pettersen (Haukeland University Hospital, Norway), Heather Probert (British Association for Cardiovascular Prevention and Rehabilitation, UK), David Prince (American Association of Cardiovascular and Pulmonary Rehabilitation, USA), Natalie Raffoul (National Heart Foundation of Australia, Australia), Kazem Rahimi (University of Oxford, UK), Julie Redfern (Institute for Evidence-Based Healthcare, Bond University & University of Sydney, Australia), Shreya Shrikhande (World Heart Federation, Switzerland), Sidney C Smith Jr (Heart and Vascular Center, University of North Carolina, USA), Rod Taylor (University of Glasgow, UK), Randal Thomas (Mayo Clinic Alix School of Medicine, Mayo Clinic Rochester, USA), Izabella Uchmanowicz (Association of Cardiovascular Nursing and Allied Professions, Poland), Joseph Weddell (University of Sydney, Australia), David Wood (National Institute for Prevention and Cardiovascular Health & University of Galway, Republic of Ireland), Hank Wu (American Association of Cardiovascular and Pulmonary

Rehabilitation, USA), Tee Joo Yeo (Singapore Heart Foundation, Singapore). We also thank Dr Ling Zhang who supported and managed the ethics application for the survey and Global Forum.
